# Contextual elaboration shapes object recognition memory across levels of childhood adversity in healthy adults

**DOI:** 10.1038/s41598-026-53083-5

**Published:** 2026-05-18

**Authors:** Annika Hanert, Anya Pedersen

**Affiliations:** Institute of Psychology, Clinical Psychology and Psychotherapy, University of Kiel, Neufeldtstraße 4a, 24118 Kiel, Germany

**Keywords:** Contextual memory, Childhood trauma, Recognition memory, Mnemonic discrimination, Neuroscience, Psychology, Psychology

## Abstract

Childhood adversity is a known risk factor for psychopathology across the lifespan. One proposed mechanism involves long-term alterations in hippocampal memory systems, leading to disruptions in the integration of episodic memory within its contextual framework. It is therefore essential to investigate how individuals exposed to early life stress use contextual information during memory formation. We conducted an experimental study in healthy adults (*n* = 76), manipulating the depth of contextual encoding. Participants viewed object-background pairs under either contextual or object-focused conditions, followed by a surprise memory test assessing object recognition and mnemonic discrimination. Childhood adversity was measured using the Childhood Trauma Questionnaire. Contrary to our initial hypothesis, contextual elaboration did not enhance recognition performance, but appeared to increase cognitive demands during encoding. We propose that increased contextual processing shifted attentional allocation away from object-specific information, thereby limiting recognition performance. Differences in recognition outcomes across conditions may further reflect variations in the strength and discriminative value of familiarity-based memory signals under differing encoding demands. We additionally observed a descriptive association between higher levels of childhood adversity and reduced object recognition performance under object-focused encoding conditions. These findings suggest that associations between childhood adversity and memory performance may emerge in a task-dependent manner and can be situated within broader theoretical accounts emphasizing the role of contextual factors in episodic memory implicated in vulnerability to mental health outcomes following early life stress.

**Supplementary Information:**

The online version contains supplementary material available at 10.1038/s41598-026-53083-5.

## Introduction

Childhood adversity has consistently been associated with an increased risk for psychopathology across the lifespan, including depression, anxiety disorders, and post-traumatic stress disorder (PTSD)^1–3^. One proposed mechanism underlying this vulnerability involves long-lasting alterations in memory-related cognitive processes, particularly those supported by stress-sensitive brain regions such as the hippocampus^4–8^. Evidence from both human and animal studies indicates that early life stress is linked to structural and functional changes in the hippocampus including reduced volume, impaired connectivity and disrupted neurogenesis in adulthood^9–11^.

The hippocampus is critically involved in episodic memory by associating items with their spatial, temporal, and semantic context into coherent memory traces^12–14^. This associative binding plays a key role in encoding and retrieval of specific autobiographical events^14,15^ and is particularly effective when contextual cues available at encoding are reinstated during recall^16,17^. When this binding fails or remains weak, the ability to retrieve detailed episodic memories can be compromised, leading to increased reliance on generalized or context-free information, a phenomenon frequently observed in trauma-exposed individuals^18,19^.

In addition to contextual binding, episodic memory critically depends on mnemonic discrimination, that is, the ability to distinguish between highly similar but non-identical events^20,21^. This process depends on pattern separation, a hippocampal neural computation supporting the transformation of similar information into distinct, non-overlapping representations, thereby reducing interference during retrieval^21–25^. Deficits in this process have been found across a range of neurological and psychiatric conditions^26–28^ and may contribute to overgeneralization of fear and maladaptive emotion regulation, particularly in PTSD and anxiety disorders^29^. Given that early adversity may compromise hippocampal integrity, assessing mnemonic discrimination may provide critical insight into how childhood stress alters memory specificity and generalization, potentially shaping cognitive vulnerability to later psychopathology.

Neuroimaging studies support this notion, showing smaller hippocampal volumes in individuals exposed to trauma^30,31^, linked to functional deficits in autobiographical memory specificity^32^ as well as contextual memory^33,34^. Importantly, such deficits in hippocampus-dependent processes are not restricted to clinical populations. Even in healthy adults, early adversity has been linked to subtle impairments in autobiographical memory^35^, pattern recognition^36^, and visual memory, mediated by reduced hippocampal connectivity^37^.

Although studies suggest that childhood trauma disrupts hippocampal memory functions, it remains unclear how these alterations affect the use of contextual information in memory processing in adulthood. While contextual elaboration typically facilitates memory retrieval through enhanced associative binding^16,38^, its effectiveness likely depends on task demands and the extent to which contextual information supports hippocampus-dependent processing, which may vary as a function of early life stress. Importantly, the use of contextual information in memory is not solely determined by hippocampal integrity, but also by the processing demands imposed during encoding, which may influence how efficiently contextual and item-specific information are integrated into memory representations^39,40^.

The present study aims to address this gap by examining how the depth of contextual encoding influences object recognition memory and mnemonic discrimination in healthy adults with varying levels of childhood adversity. Using a between-subjects design, we systematically manipulated encoding depth by instructing participants either to semantically elaborate object-context pairings or to encode the same stimuli without direct elaboration of the background context. At retrieval, memory performance was assessed under context representation. Additionally, we examined whether individual differences in childhood adversity moderate the effect of context processing on memory outcome.

Based on previous research, we hypothesized that higher levels of childhood adversity would be associated with poorer hippocampus-dependent memory performance^9,41^, as reflected in reduced object recognition accuracy and mnemonic discrimination. Furthermore, we expected that contextual elaboration would enhance memory performance overall by promoting associative binding^38^. Finally, we examined whether contextual elaboration interacts with childhood adversity, such that it may either compensate for underlying memory deficits by providing external support or exacerbating them when hippocampal dysfunction impairs effective binding.

## Methods

### Participants

A total of 94 participants (24.22 ± 3.87 years, 18–36 range, 71 female) were recruited from the University of Kiel and the surrounding community. To motivate participation, a prize draw offering vouchers was conducted. Additionally, students who completed the assignment could receive course credit. Individuals between the ages of 18 and 40 and with German as mother language were eligible for inclusion. Those with a current diagnosis of a psychiatric disorder or who were taking medications known to affect memory or attentional processes were excluded from participation. The study was approved by the Ethical Committee of the Faculty of Medicine at the University of Kiel, Germany, which also approved the informed consent procedure, under approval number D590/23. All procedures were in accordance with the Declaration of Helsinki. The study was conducted within the laboratory facilities of the Department of Psychology at the University of Kiel. Before taking part, all participants were given both written and verbal explanations of the study, had the opportunity to ask questions, and then provided their written informed consent.

## Materials and Procedure

### Memory paradigm and experimental conditions

**Fig. 1:**
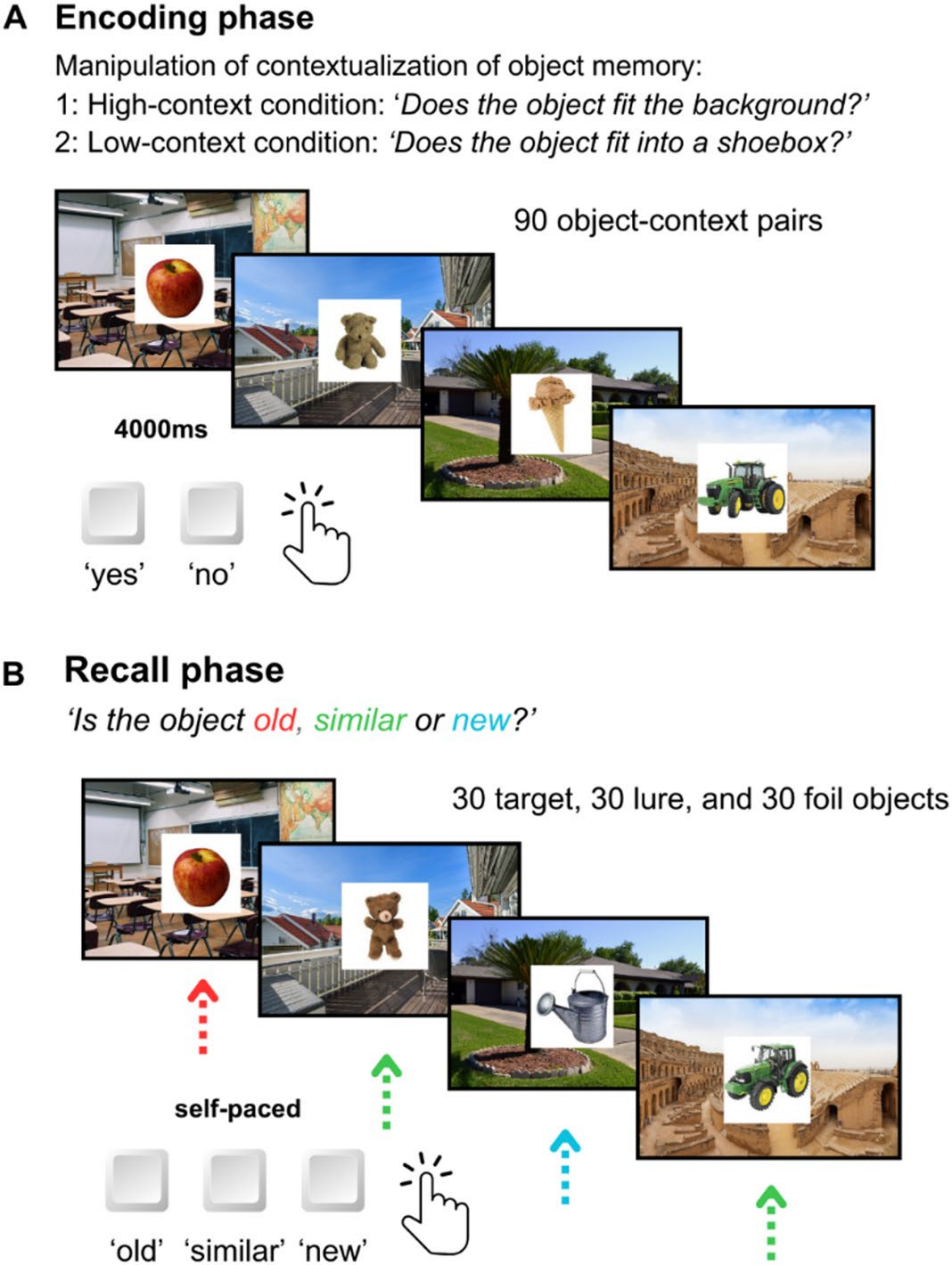
Overview of the Contextual Object Recognition Test and experimental manipulation. A) Participants encoded 90 object-context pairs in one of two experimental conditions: In the high-context condition, they judged whether the object fits the background, whereas in the low-context condition, they judged whether the object fits inside a shoebox. (B) In the surprise recall phase, participants were presented with 30 previously seen objects (targets), 30 visually similar but not identical objects (lures), and 30 entirely novel objects (foils). They were required to classify each object as ‘old’, ‘similar,’ or ‘new’. Answers were all given via button press.

The paradigm was designed as a Contextual Object Recognition Test (CORT) based on the widely used Mnemonic Similarity Task (MST)^42^. During each trial of the encoding phase, participants were presented with visual stimuli on a computer screen. Those comprised a colored photograph superimposed on a background image depicting different contextual environments, creating an object-context pairing. The objects displayed were photographs of everyday objects (e.g., tools, toys or food) taken from the MST. The background scenes consisted of photographs shot from a first-person perspective, simulating a naturalistic viewpoint of the environment (see Fig. 1). The scenes were balanced across different environmental domains to ensure diversity in contextual representation. These domains encompassed private spaces (e.g., living room), recreational settings (e.g., movie theater), work-related environments (e.g., office scene), public spaces (e.g., airport), and natural landscapes (e.g., forest). The background images were obtained from the freely available Pixabay database (www.pixabay.com).

Participants were randomly assigned to one of the two experimental conditions, a high-context and a low-context condition. During the encoding phase, participants were presented with a total of 90 unique object-context pairs. The sole distinction between the conditions pertained to the question participants responded to during the encoding phase, which was designed to manipulate the degree of object contextualization during object-context encoding. In the high-context condition, the question ‘Does the object fit the background?’ aimed for a higher degree of contextualization, inducing a semantic elaboration of both stimuli, while the question ‘Does the object fit into a shoebox?’, in the low-context condition, was placed to maintain a similar depth of memory processing by not recuring to the background context, but focusing on the object. ‘Yes’- or ‘No’-Responses were provided via button press on a keyboard. Encoding duration was recorded as the time (s) from the appearance of the stimulus to the button press, and used to measure task engagement.

In the subsequent surprise recall phase, following the procedure of the MST, participants were presented with object-context pairs in which the objects were either identical to previously encoded objects (targets), visually similar but not identical (lures), or completely novel (foils). For each object-context pair the background scene remained consistent between the encoding and recall phases, thus ensuring that lures were presented within the same contextual setting as their corresponding targets. Additionally, novel objects were displayed on previously encoded background scenes; however, in these cases the original object associated with that background during encoding was not shown again. Of the previously encountered object-context pairs, 30 were presented as old targets, 30 as visually similar lures, and 30 as novel foils. Participants were instructed to identify these objects as ‘old,’ ‘similar’, or ‘new” using corresponding buttons. Each object-context pair was presented self-paced; the stimulus remained on screen for 4 seconds, followed by a 1-second interval initiated after the response. In both conditions all stimuli were presented in a random sequence. The experimental paradigm was presented using PsychoPy v2022.2.4.

Recognition memory performance as well as lure discrimination were calculated in line with previous studies^42^ as the hit rate of targets and lures, respectively, corrected for false alarms (i.e. recognition memory = p(‘old’ | target) - p(‘old’ | foil); lure discrimination = p(‘similar’ | lure) - p(‘similar’ | foil)). In order to ensure a reliable calculation of these memory indices and to include only participants who demonstrated sufficient engagement with the task, a performance-based learning criterion was applied prior to statistical analysis. Participants were excluded if they (a) showed recognition accuracy below the chance level of 33% (i.e., the proportion of ‘old’ responses to targets), (b) had a lure discrimination score below zero, or (c) demonstrated an extreme response bias toward ‘old’ responses, operationalized as giving more than 30% ‘old’ responses to foil items. These criteria served to identify individuals who likely did not encode the stimuli meaningfully or failed to adhere to task instructions. Based on these predefined thresholds, 18 participants were excluded from the final analysis (n = 10 in the low-context condition, n = 8 in the high-context condition). The resulting sample comprised 76 participants (n = 37 in the low-context condition, n = 39 in the high-context condition).

### Assessment of childhood adversity

As the outcome measure of early adverse events, the Childhood Trauma Questionnaire-Short Form (CTQ-SF)^43^ was used. The CTQ is a self-report measure developed to assess adverse childhood experiences. The questionnaire is divided into five subscales: (1) physical abuse, (2) sexual abuse, (3) emotional abuse, (4) physical neglect, and (5) emotional neglect. The instrument comprises 28 items, each beginning with the phrase ‘When I was growing up’, followed by statements such as ‘I didn’t have enough to eat.’ (physical neglect) or ‘I thought my parents wished I had never been born.’ (emotional abuse). Participants respond on a five-point Likert scale, ranging from ‘Never true’ to ‘Very often true’. The total score ranges from 25, indicating no history of childhood trauma, to 125, indicating severe trauma exposure^43^.

### Mental health questionnaires

As a component of a broader set of surveys, participants provided information about demographics and mental health condition. The survey included the subscales of the Patient Health Questionnaire (PHQ-9) and the Generalized Anxiety Disorder scale (GAD-7)^44^. Additionally, participants provided responses of the Beck Depression Inventory-II (BDI-II)^45^. These measures were administered to characterize the sample, as the study targeted a non-clinical population. Participants in both conditions did not differ regarding age, gender, and mental health status (Table 1).

**Table 1:** Demographic data and questionnaire results between groups.

	High-context (*n* = 39)	Low-context (*n* = 37)	*p*
Age (Mean ± SD, range)	23.74 ± 3.84, 18–34	24.73 ± 3.81, 19–35	0.265
Gender	28 female	29 female	.346^a^
BDI-II (Mean ± SD)	8.56 ± 6.01	7.46 ± 4.87	0.383
PHQ-9 (Mean ± SD)	6.28 ± 3.53	5.84 ± 3.39	0.578
GAD-7 (Mean ± SD)	5.00 ± 3.74	4.92 ± 3.29	0.930

Note. Comparisons between conditions were conducted using independent-samples t-tests for continuous variables and ^a^Fisher’s exact test for categorical variables. Abbreviations: BDI-II (Beck Depression Inventory II), PHQ-9 (Patient Health Questionnaire), GAD-7 (Generalized Anxiety Disorder 7).

### Statistical analyses

Statistical analyses were conducted using IBM SPSS Statistics (Version 29) and Python (Version 3.13).

For descriptive purposes, validated cut-off criteria^43^ were applied to categorize participants’ childhood trauma exposure levels. In addition, measures of variability (standard deviations and coefficients of variation (CV)) were computed to describe the distribution of CTQ subscale scores in the present non-clinical sample.

To examine the effects of childhood adversity on memory performance, a series of multiple linear regression analyses was performed. The primary dependent variables were recognition memory and lure discrimination, derived from performance in the MST and analyzed at the participant level. Separate regression models were computed for each dependent variable. In each model, experimental condition (high vs. low context) and the CTQ sum score were entered as predictors. Additional exploratory models included either emotional neglect or emotional abuse as continuous predictors. To examine moderation effects, an interaction term between condition and the respective adversity variable was included in each model. Prior to conducting regression analyses, all model assumptions were assessed. Normality of residuals was tested by the Shapiro-Wilk test. Homoscedasticity was assessed using the Breusch-Pagan test. As several models revealed deviations from homoscedasticity and normality of residuals, heteroscedasticity-consistent standard errors (HC3) were used to ensure robust inference^46^. All computed Durbin-Watson values fell within the acceptable range, suggesting no substantial autocorrelation of residuals. Although the variance inflation factors used to test for multicollinearity were elevated for the models that included interaction terms, this was expected due to the inherent collinearity between the interaction terms and their components. To quantify the magnitude of moderation effects, changes in explained variance (ΔR²) were computed by comparing nested regression models with and without the interaction term. In addition, a sensitivity analysis was conducted using G*Power (linear multiple regression: fixed model, R² increase) to determine the smallest detectable interaction effect given the sample size, assuming α = 0.05 and 80% power.

To compare group differences in memory performance (recognition memory and lure discrimination) independently of adversity variables, independent samples t-tests were conducted. If the assumption of homogeneity of variances (tested by Levene’s test) was violated, the Welch correction was applied.

Encoding duration was analyzed at the trial level to assess both overall differences between context conditions and its potential role in shaping subsequent memory performance. Differences in encoding duration between the high-context and low-context conditions were examined using a linear mixed-effects model with trial-level encoding duration as the dependent variable and random intercepts for participants. To test whether encoding duration modulated memory accuracy, mixed-effects logistic regression models were fitted at the trial level. To examine trial-level response behavior, generalized linear mixed-effects models with a logit link function were applied. For each item category (targets, lures, foils) and each response type (old, similar, new), a separate model was estimated, with the binary recall response (correct vs. incorrect) as the dependent variable. Encoding duration was entered as a z-standardized continuous predictor, alongside encoding condition (high vs. low-context) and their interaction. All trial-level models included random intercepts for participants and trials to account for repeated observations across trials within participants and item-specific variability. For linear mixed-effects models, residual distributions and residuals versus fitted values were inspected visually. For generalized linear mixed-effects models, model convergence and dispersion were evaluated using diagnostic checks.

An alpha level of 0.05 (two-tailed) was used for all inferential tests, unless Bonferroni correction was applied where appropriate; adjusted alpha levels were explicitly indicated in the relevant results. For regression models, effect sizes were reported as R², and for individual predictors, unstandardized coefficients (B), standardized coefficients (β), and 95% confidence intervals were provided. For mixed-effects models, fixed effects were reported as unstandardized coefficients (B) or log odds ratios, together with standard errors, test statistics, and p values. Random effects were included to account for subject- and item-level variability but are not reported in detail.

### Use of generative AI in the writing process

OpenAI’s ChatGPT (version GPT-4, OpenAI, San Francisco, CA, USA) was used to assist in refining the language and comprehensibility of the manuscript, as well as optimization of Python codes for data analysis and visualization. All content was subsequently reviewed and edited by the authors, who take full responsibility for the final version of the article.

## Results

### Between-group differences in memory performance

**Fig. 2:**
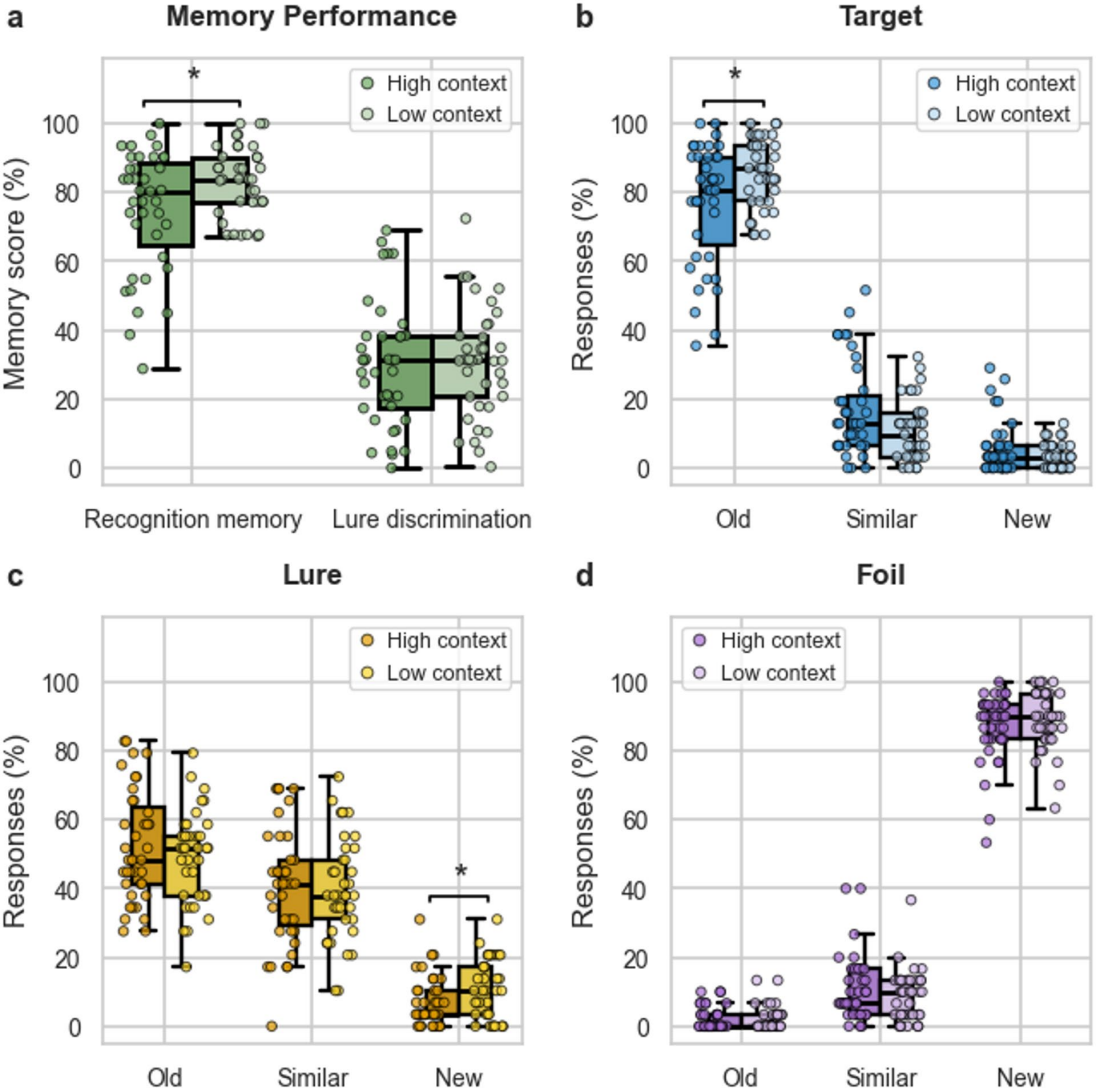
Distribution of memory performance as a function of contextual condition (low vs. high). Individual data points are overlaid on boxplots to illustrate within-group variability. (a) Results of the calculated memory scores ‘recognition memory’ and ‘lure discrimination’ in the high- and low-context conditions. There was a significantly better performance regarding recognition memory in the low-context condition. (b) Mean response proportion regarding targets with a significantly higher correct response in the low-context condition. (c) Mean response proportion regarding lures; in the high-context condition there was a significantly higher false response (i.e. declaring lures as ‘new’). D) No difference between the two conditions regarding responses to foils. For all between-group comparisons involving response types (‘old’, ‘similar’, ‘new’), Bonferroni correction was applied (k = 3), yielding an adjusted significance level of α = 0.0167. * *p* < .0167.

First, we investigated the differences in recognition memory performance and lure discrimination between the two conditions. A clear difference emerged in recognition memory (t(61.31) = 2.64, *p* = .011, 95% CI [2.10, 15.25], d = 0.69), with significantly higher performance in the low-context condition (M = 83.54, SD = 10.18) compared to the high-context condition (M = 74.87, SD = 17.68). No significant difference was found between conditions regarding lure discrimination (t(74) = 0.20, *p* > .05, 95% CI [-7.03, 8.61], d = 0.05, Fig. 2a). Second, we analyzed the response behavior to targets, lures, and foils, respectively. Regarding target items (Fig. 2b), a Welch’s t-test revealed a significant difference in correct responses to targets (i.e. correctly identifying targets as ‘old’; t(60.87) = 2.70, *p* = .009, 95% CI [2.25, 15.15] d = 0.61). Again, participants in the low-context condition performed significantly better (M = 85.79, SD = 9.90) than those in the high-context condition (M = 77.09, SD = 17.40). With regard to lure items (Fig. 2c), a Student’s t-test showed a significant group difference in the proportion of false responses (i.e. ‘new’ response; t(74) = 2.21, *p* = .030, 95% CI [0.38, 7.47], d = 0.57). Participants in the high-context condition were less likely to incorrectly classify lure items as ‘new’ (M = 7.07, SD = 7.07) than the low-context condition (M = 11.00, SD = 8.40). For the foil items (Fig. 2d), no significant differences were found in the individual responses (all p’s > 0.05, see also Table S1 for additional characteristics of inferential statistical analysis).

Third, we examined whether yes vs. no judgements during encoding were associated with differences in trial-level memory performance between conditions using generalized linear mixed-effects models for targets, lures, and foils. The interaction between judgment (yes vs. no) and condition was not significant for any item category (targets: β = 0.11, SE = 0.26, z = 0.43, *p* = .668, 95% CI [-0.40, 0.63]; lures: β = -0.38, SE = 0.26, z = -1.49, *p* = .137, 95% CI [-0.88, 0.12]; foils: β = -0.27, SE = 0.32, z = -0.87, *p* = .386, 95% CI [-0.89, 0.34]).

### Exploratory analysis of encoding duration and effects on response during recall

As an exploratory step, encoding duration was examined to assess whether prolonged engagement during encoding might be associated with subsequent memory performance. First, a linear mixed-effects model was fitted with condition (high- vs. low-context) as a fixed effect and random intercepts for participants and trials to account for the hierarchical structure of the data. Encoding duration was significantly longer in the high-context condition compared to the low-context condition (β = 0.25, SE = 0.09, t = 2.94, *p* = .004).

Second, to investigate whether encoding duration was associated with subsequent response behavior at test, generalized linear mixed-effects models (hierarchical logistic regressions) were computed. Models were estimated separately for each item category (targets, lures, foils) and each response type (old, similar, new). Encoding duration (z-standardized), condition, and their interaction were included as predictors. Model estimates therefore reflect changes in the probability of a specific response type as a function of encoding duration and condition.

For target items, a significant main effect of condition was observed for old responses. Participants in the high-context condition showed a lower probability of correctly identifying targets as ‘old’ compared to the low-context condition (β = −0.66, SE = 0.25, z = − 2.64, p = 0.008, OR = 0.52, 95% CI [0.32, 0.84]), which remained significant after Bonferroni correction (k = 3, α = 0.017). Encoding duration was not significantly associated with’ old’ responses to targets (β = −0.05, SE = 0.10, z = − 0.49, *p* = .624), and the interaction between encoding duration and condition did not reach significance (*p* = .055).

A comparable pattern was observed for ‘similar’ responses to targets, with a significant main effect of condition indicating a higher probability of ‘similar’ responses in the high-context condition (β = 0.63, SE = 0.24, z = 2.61, *p* = .009, OR = 1.87, 95% CI [1.17, 3.00]), which also survived Bonferroni correction. Encoding duration and the interaction term were not significant (all p’s > 0.11). No significant effects were observed for ‘new’ responses to targets (all p’s > 0.249).

For lure items, a main effect of condition was observed for ‘new’ responses, indicating a lower probability of responding new in the high-context condition (β = −0.56, SE = 0.24, z = − 2.32, *p* = .020, OR = 0.57, 95% CI [0.36, 0.92]), although this effect did not survive Bonferroni correction. Importantly, a significant interaction between encoding duration and condition emerged for ‘new’ responses to lures (β = −0.69, SE = 0.22, z = − 3.15, *p* = .002, OR = 0.50, 95% CI [0.33, 0.77]), which remained significant after Bonferroni correction. This interaction indicates that longer encoding duration was associated with a reduced likelihood of new responses to lures specifically in the high-context condition. No significant effects were found for ‘old’ or ‘similar’ responses to lures (all p’s > 0.171).

For foil items, none of the models revealed significant effects (all p’s > 0.221). All parameter estimates from the generalized linear mixed-effects models are reported in Table S4 in the Supplementary Materials.

### Effects of childhood adversity and context manipulation on memory performance

#### CTQ scores and prevalence of childhood adversity

To characterize the extent of childhood adversity in our sample, descriptive statistics were computed for all CTQ subscales (see Table 2). As expected, there were no significant differences between experimental conditions regarding either subscale scores or prevalence rates of participants who met or exceeded the ‘low to moderate’ threshold based on Bernstein et al.^43^(see Table S2; all p’s > 0.05). To evaluate the interpretability of the subscale scores with regard to potential floor effects, we further assessed their relative variability. Here, the coefficient of variation (CV) was calculated for each subscale across both conditions. *Emotional abuse* (CV = 43.00%) and *emotional neglect* (CV = 37.96%) showed the highest relative variability, followed by *physical neglect* (CV = 30.86%), *sexual abuse* (CV = 26.52%), and *physical abuse* (CV = 17.64%). Thus, emotional neglect and emotional abuse exhibited the strongest interindividual differentiation in our preclinical sample. To further demonstrate their validity, we analyzed the intercorrelation at the sample-based level. Emotional neglect and emotional abuse were significantly correlated (*r* = .643, *p* < .001), indicating related but not independent dimensions of emotional maltreatment. Given this intercorrelation, subscale analyses were treated as exploratory, and primary conclusions were based on the CTQ sum score.

**Table 2:** Descriptive and inferential statistics for CTQ scores between conditions.

CTQ	High-context M ± SD (Min – Max)	Low-context M ± SD (Min – Max)	t(df)	*p*	95%CI
Emotional neglect	9.33 ± 2.98 (5–16)	9.54 ± 4.15 (5–19)	0.25 (74)	0.803	[-1.44,1.85]
Emotional abuse	8.41 ± 3.31 (5–16)	9.05 ± 4.18 (5–23)	0.74 (74)	0.458	[-1.08,2.36]
Physical abuse	5.18 ± 0.39 (5–6)	5.49 ± 1.28 (5–12)	1.40 (42.24)	0.170	[-0.14,0.75]
Physical neglect	6.28 ± 1.65 (5–11)	7.22 ± 2.38 (5–13)	1.98 (63.81)	0.052	[-0.01,1.88]
Sexual abuse	5.56 ± 1.55 (5–13)	5.59 ± 1.42 (5–11)	0.09 (73.92)	0.929	[-0.65,0.71]
Sum Score	34.77 ± 6.50 (26–50)	36.89 ± 10.45 (25–66)	1.06 (59.64)	0.295	[-1.89,6.14]

Note. The subscales are all measured on a scale ranging from 5 to 25. Consequently, the total score ranges from 25 to 125.

### Influence of childhood adversity on recognition memory

**Fig. 3:**
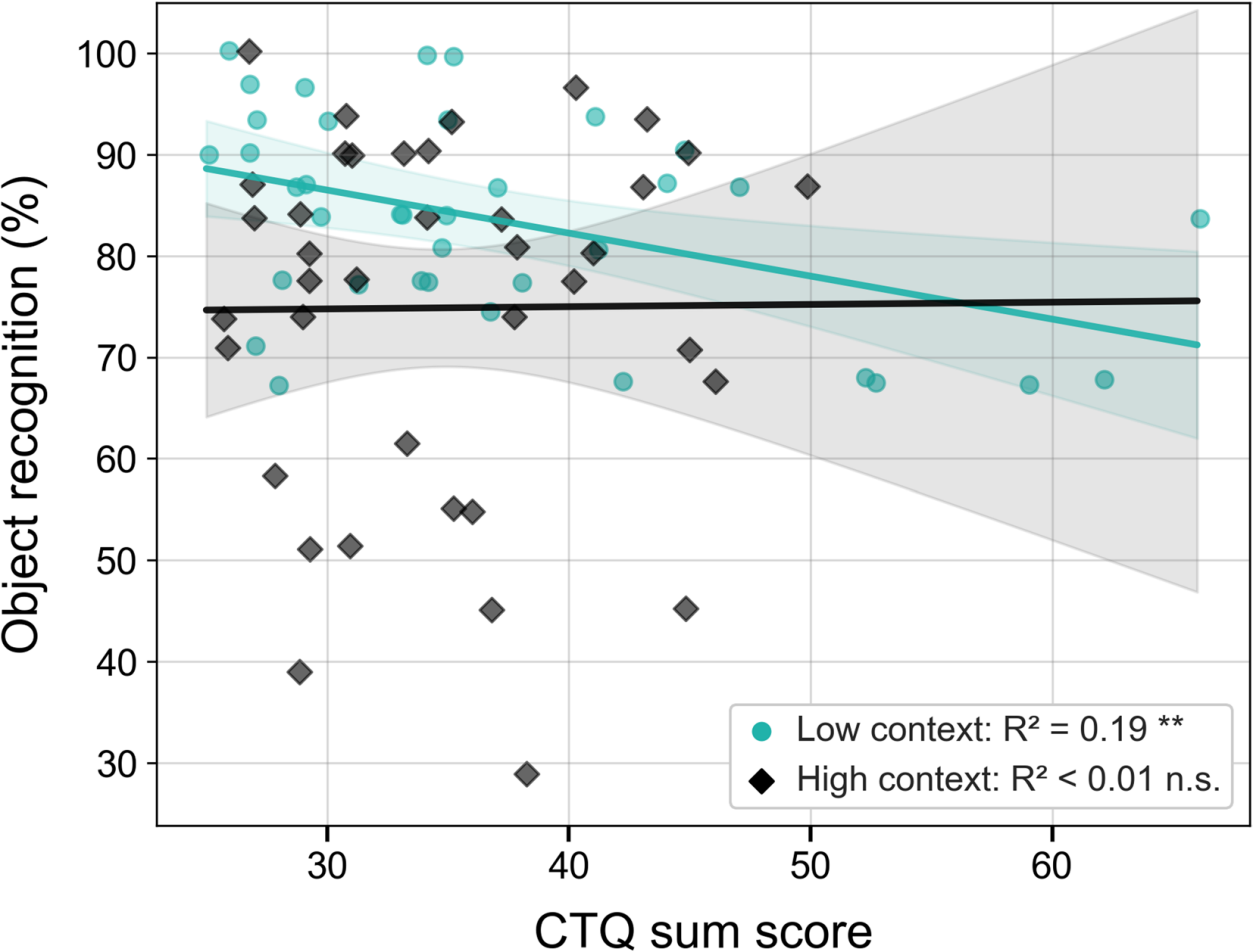
Scatter plots depicting the relationship between the CTQ sum score and object recognition performance. There was a significant correlation in the low-context condition (**p < .01). Slopes represent fitted regression lines for each encoding condition and are shown for descriptive purposes only, as the corresponding interaction effects were not statistically significant. Shaded areas indicate 95% confidence intervals. Additional scatter plots including regression slopes for the lure discrimination index and CTQ scores (total score, emotional neglect, and emotional abuse) are provided in Figure S1 in the Supplementary Material for transparency.

To examine whether childhood adversity predicted object recognition, a series of linear regression analyses were conducted. Childhood adversity was operationalized using the CTQ sum score as a composite index, as well as the emotional neglect and emotional abuse subscales, which were examined exploratorily. Each model included condition (context group: low vs. high) and the respective CTQ measure as predictors, and the interaction term between group and CTQ score. Full regression results, including models with and without interaction terms, are reported in Table S3.With regard to the impact of overall childhood adversity (CTQ sum score), the model was statistically significant, F(3, 72) = 4.37, *p* = .007, explaining approximately 12.6% of the variance in recognition memory performance. The CTQ sum score showed a significant negative association with recognition memory (B = -0.43, SE = 0.18, β =-0.25, t=-2.43, *p* = .017, 95% CI [-0.78, -0.08]). The main effect of condition was not significant (B = -25.47, SE = 16.06, β = − 0.85, t= -1.59, *p* = .117, 95% CI [-57.48, 6.55]), and there was no significant interaction between the CTQ sum score and condition (B = 0.46, SE = 0.46, β = 0.55, t = 1.00, *p* = .319, 95% CI [-0.45, 1.36]). To further explore the main effect of childhood adversity measured by the CTQ sum score on recognition memory, analyses of simple slopes were conducted separately for each condition. To illustrate the association between childhood adversity and recognition memory within each condition, simple slopes are shown for descriptive purposes. In the low-context condition, the slope was negative (B = -0.43, SE = 0.18, t(72) = -2.43, *p* = .015), whereas in the high-context condition the slope was close to zero (B = 0.03, SE = 0.42, t(72) = 0.07, *p* = .944; Fig. 3).

Regarding emotional neglect and recognition memory the overall model was statistically significant, F(3, 72) = 4.71, *p* = .005, explaining 12.8% of the variance in recognition memory (R² = 0.128). We also found a significant main effect of emotional neglect, suggesting that higher levels of adversity were associated with lower recognition memory performance (B = -1.05, SE = 0.40, β = − 0.25, t(72) = -2.64, *p* = .010, 95% CI [-1.84, -0.26]). Moreover, there was a significant main effect of condition, indicating that participants in the low-context condition showed better recognition memory performance than those in the high-context condition (B = -22.87, SE = 9.36, β = − 0.58, t(72) = -2.44, *p* = .017, 95% CI [-41.54, -4.21]). We did not find a significant interaction (B = 1.50, SE = 0.97, β = 0.36, t(72) = 1.55, *p* = .125, 95% CI [-0.44, 3.42]). To further explore the association between emotional neglect and recognition memory within each encoding condition, separate regression slopes were calculated within each group for descriptive purposes. In the low-context condition, the slope was negative (B = -1.05, SE = 0.40, t(72) = -2.64, *p* = .008). In the high-context group, the slope was positive but small (B = 0.45, SE = 0.88, t(72) = 0.51, *p* = .609; Figure S1c).

The overall model examining emotional abuse and recognition memory was statistically significant, F(3, 72) = 4.44, *p* = .006, explaining 11.2% of the variance (R² = 0.112). A significant main effect of emotional abuse emerged, indicating that higher levels of emotional abuse were associated with lower recognition memory performance (B = -0.87, SE = 0.36, β = − 0.22, t(72) = -2.45, *p* = .017, 95% CI [-1.58, -0.16]). Analyses revealed no significant main effect of condition (B = -16.47, SE = 8.39, β = − 0.60, t(72) = -1.96, *p* = .054, 95% CI [-33.21, 0.26]). The interaction between condition and emotional abuse was also not statistically significant (B = 0.86, SE = 0.91, β = 0.21, t(72) = 0.95, *p* = .346, 95% CI [-0.95, 2.67]). To further illustrate the association between emotional abuse and recognition memory, separate regression slopes are presented for descriptive purposes. In the low-context condition, the slope was negative (B = -0.87, SE = 0.36, t(72) = -2.44, *p* = .015), whereas in the high-context condition the slope was close to zero (B = -0.01, SE = 0.84, t(72) = -0.01, *p* = .991; Figure S1e).

### Influence of childhood adversity on lure discrimination

To examine whether childhood adversity also predicted lure discrimination performance, further linear regression models were conducted using the CTQ sum score as the primary predictor and emotional neglect and emotional abuse as exploratory predictors. Each model included condition (context group: low vs. high) the respective CTQ measure, and the interaction term between condition and CTQ score. A comprehensive overview of all regression parameters is provided in Table S3.

The overall model regarding the CTQ sum score was not statistically significant, F(3, 72) = 0.21, *p* = .892, and explained only 1.1% of the variance in lure discrimination (R² = 0.011). None of the predictors showed significant effects. The CTQ sum score was not significantly associated with lure discrimination (B = -0.22, SE = 0.32, β = − 0.11, *p* = .498, 95% CI [-0.85, 0.42]). Similarly, the main effect of condition (B = -13.78, SE = 20.00, β = − 0.41, *p* = .493, 95% CI [-53.64, 26.09]) and the interaction term (B = 0.36, SE = 0.56, β = 0.38, *p* = .519, 95% CI [-0.75, 1.47]) were not statistically significant.

The overall regression model including emotional neglect, condition, and their interaction was not significant (F(3, 72) = 0.44, *p* = .728 R² = 0.017). No significant effects emerged for emotional neglect (B = -0.67, SE = 0.65, β = − 0.14, t(72)= -1.03, *p* = .305), group (B = -2.79, SE = 12.50, β = − 0.05, t(72)= -0.22, *p* = .824), or the interaction term (B = 0.20, SE = 1.31, β = 0.05, t(72), *p* = .880). Thus, emotional neglect was not associated with lure discrimination performance, regardless of encoding context. A similar pattern of results was shown for the model examining emotional abuse, encoding group (high- vs. low-context), and their interaction, which did not reach overall statistical significance (F(3, 72) = 2.10, *p* = .108, R² = 0.072). Thus, we did not further interpret the interaction term (B = 2.45, SE = 1.00, β = 0.54, t(72) = 2.46, *p* = .016, 95% CI [0.46, 4.43]) as well as the main effects of emotional abuse (B = -0.77, SE = 0.64, β = − 0.17, t(72) = -1.21, *p* = .231, 95% CI [-2.05, 0.50]) and group (B = -21.86, SE = 10.0, β = − 0.03, t(72) = -2.20, *p* = .031, 95% CI [-41.70, -2.01]).

### Sensitivity analysis and magnitude of observed interaction effect

A sensitivity analysis using G*Power (Version 3.1.9.7) indicated that, given the present sample size (*N* = 76), α = 0.05 and (1-β) = 0.80, the regression models were sufficiently powered to detect moderation effects of small-to-moderate magnitude (Cohen’s f² ≈ 0.10). To quantify the magnitude of the observed interaction effects, changes in explained variance (ΔR²) were computed by comparing nested regression models with and without the interaction term. The observed interaction effects were small for recognition memory (ΔR² = 0.01 to ΔR² = 0.03) and negligible for lure discrimination (ΔR² = 0.001 to ΔR² = 0.007), indicating that any moderation effects were below the level reliably detectable with the current design. Observed ΔR² values for all regression models are reported in Supplementary Table S3.

## Discussion

Childhood adversity has been associated with long-lasting alterations in memory performance, particularly under conditions that increase processing demands on declarative encoding and retrieval^36,37,47^. Against this backdrop, this study examined how object recognition memory and mnemonic discrimination are shaped by contextual elaboration at encoding in individuals with varying degrees of experienced stress during childhood. We found that object recognition memory performance was diminished when encoding emphasized contextual information, in contrast to our initial hypothesis that contextual elaboration would facilitate memory performance. Critically, childhood adversity as measured by the CTQ was negatively associated with object recognition memory in the low-context condition. Contrary to our assumption, mnemonic discrimination was not significantly influenced by context-specific manipulations nor systematically impaired by early adversity. Together, these results delineate distinct patterns of object recognition memory and mnemonic discrimination in relation to contextual memory processing and childhood adversity even in healthy individuals.

Our findings indicate that increased contextual elaboration during encoding can impair, rather than facilitate, object recognition memory. The encoding specificity principle posits that memory retrieval is most effective when the contextual cues available at encoding are reinstated at test^17^, as this overlap facilitates access to stored representations. However, in the present paradigm, object recognition was superior when encoding placed fewer demands on contextual processing, suggesting that contextual information may have interfered with, rather than supported, object-related information. This pattern can be interpreted in the light of accounts emphasizing attentional competition between item and contextual information. According to the outshining effect, highly salient item-specific information can dominate retrieval processes and render contextual cues ineffective or even distracting^38,48,49^. Conversely, the overshadowing effect proposes that attention directed to background information during encoding can weaken item processing, resulting in poorer recognition performance^50,51^. Together, these accounts point to attentional competition during encoding as a critical factor shaping object recognition, a notion that is further supported by our analyses of encoding behavior.

Further support for this attentional account comes from the observation that encoding duration was significantly longer in the high-context condition, indicating increased processing demands during encoding^40^. However, extended encoding duration did not improve recognition of target items. This dissociation suggests that the high-context manipulation increased attentional allocation during encoding, likely distributing processing across both the object and its surrounding scene rather than sharpening object-specific representations^52^. In this sense, the present findings are consistent with broader accounts of divided attention during encoding, which show that allocating attention across multiple information sources can impair the formation of item-specific memory representations^39,40^. Consistent with this interpretation, increased perceptual and integrative processing demands during encoding have been shown to prolong encoding time while impairing subsequent object recognition, suggesting a shift toward stimulus integration rather than object-specific strengthening^53^.

At the behavioral level, longer encoding duration in the high-context condition was associated with a reduced likelihood of responding ‘new’ to similar lure items, indicating that lures were less often experienced as novel. This selective effect suggests that extended processing time did not strengthen object recognition per se, but instead increased the availability of a general memory signal, likely familiarity-based, that guided responses toward ‘old’ or ‘similar’ classifications. Consistent with dual-process models, longer encoding duration may have increased the overall strength of familiarity-based signals without sharpening object-specific representations, thereby biasing responses toward ‘old’ or ‘similar’ judgments without improving target recognition^54,55^. Crucially, encoding-related Yes/No judgments were not associated with subsequent object recognition performance. Thus, the outcome of the semantic evaluation itself did not confer a recognition advantage. Instead, the data indicate that recognition outcomes were shaped by the allocation of attentional resources during encoding as indexed by longer processing durations rather than by the semantic evaluation of object-background relations.

While our findings are consistent with an attentional account emphasizing competition between object and contextual information during encoding, alternative explanations should also be considered. In addition to attentional factors, it is possible that the contextual information provided in the high-context condition was not diagnostic for successful object recognition at retrieval. Moreover, as the recall task required object-based judgments (i.e. ‘old’/‘similar’/‘new’) and did not assess contextual memory, participants’ attention at retrieval was explicitly directed toward the objects themselves, limiting conclusions about whether contextual information was reinstated or used as a retrieval cue. Accordingly, although contextual information was processed during encoding, it did not reliably support later recognition decisions, consistent with prior work showing that contextual or relational information can be encoded and retained without contributing diagnostic evidence for item recognition^13,17,38^.

Another relevant observation of our study concerns the association between childhood adversity and recognition performance within the low-context condition. Although the interaction between encoding condition and adversity was not statistically significant, we observed a descriptive association indicating that higher levels of childhood adversity were associated with reduced recognition performance when contextual support at encoding was reduced. Importantly, this pattern should not be interpreted as evidence for differential effects across encoding conditions, but rather as an association that was more apparent under low-context encoding. Given the limited statistical power to detect interactions, it is possible that such associations were not reliably detectable across conditions. A sensitivity analysis indicated that the present sample size provided sufficient power to detect moderation effects of small-to-moderate magnitude. However, the observed interaction effects were consistently small for recognition performance and negligible for lure discrimination, falling below the effect sizes that could be reliably detected with the current design. These findings suggest that potential context-dependent effects of childhood adversity, if present, are likely subtle at the behavioral level.

One cautious interpretation is that under low-context encoding, where recognition relies more strongly on object-focused information and fewer contextual cues, individual differences related to childhood adversity may become more readily observable, because recognition relies more directly on object-based memory signals, with limited contextual cues. In contrast, the availability of additional contextual information in the high-context condition may have reduced the sensitivity of the task to detect such subtle associations, without implying that the underlying effect of vulnerability differs across conditions. Consistent with this view, prior research suggests that the cognitive impact of childhood adversity on memory performance is not uniform, but varies as a function of task demands and processing requirements, also in non-clinical populations^56,57^. Notably, situating the present findings within a broader literature, prior research indicates that childhood adversity is associated with subtle alterations across multiple memory domains, particularly under conditions that place higher demands on representational specificity. These effects have been observed in autobiographical memory^32,58,59^, in associative memory tasks involving emotionally salient or threat-related cues^41,56^, and in visuo-spatial recognition memory^36^. Although these paradigms differ from the present object recognition memory task, they converge in highlighting task conditions under which memory performance is more vulnerable to the effects of early adversity.

From a theoretical perspective, models linking childhood adversity to long-term alterations in memory-related processes provided the initial motivation for the present study^6,60^. Such accounts are often discussed with reference to stress-related alterations in medial temporal lobe systems, with a special focus on the hippocampus, which persist into adulthood and compromise declarative memory performance^61–64^. Within theoretical models distinguishing object-based familiarity from relational and contextual memory, memory processes that rely primarily on object-focused information have often been discussed in relation to perirhinal cortex, whereas hippocampal processes are more commonly associated with relational and contextual aspects of memory^13,65,66^. Beyond perirhinal contributions to item-based representations, converging evidence suggests that the parahippocampal cortex supports the representation of contextual information, particularly the spatial and environmental aspects of an experience, as reflected in its preferential response to scenes and spatial layouts over objects^12,67^, while also contributing to more abstract, non-spatial aspects of context^13,68^. Within this framework, the hippocampus is thought to bind item- and context-related information into integrated episodic representations^14,69^. In the present paradigm, the high-context condition may therefore have promoted the encoding of richer item-context representations, consistent with the notion that increased processing demands during encoding led to a broader allocation of attentional resources across both object and contextual information^39,52^. This shift may have involved greater engagement of context-processing mechanisms typically associated with the parahippocampal cortex^12,70^, resulting in more integrated representations that include both object and contextual features, rather than selectively strengthening object-specific information. Although the present study does not directly assess such mechanisms, the observed pattern may be consistent with the notion that contextual information, while processed during encoding, does not effectively support subsequent memory decisions, potentially reflecting reduced efficiency in the integration of contextual and item-specific information. However, as the current findings are based exclusively on behavioral measures, such frameworks should be regarded as conceptual rather than mechanistic accounts. Accordingly, the present results are best interpreted as behavioral evidence for an association between childhood adversity and recognition performance under conditions of reduced contextual support, without implying specific neural mechanisms.

When examining mnemonic discrimination at the group level, we did not observe a significant main effect of contextual elaboration. This finding diverges from prior studies showing that context cues can bias responses to items that share a significant overlap with previously learned ones. For instance, Racsmány et al.^71^ reported increased false alarms to lures when irrelevant backgrounds were shown, suggesting that contextual cues can impair mnemonic specificity. Similarly, Doss et al.^72^ highlighted distortions in episodic memory due to contextual overlap, particularly through increased reliance on familiarity signals. In our study, we did not observe such a statistically significant increase in false ‘old’ responses to lures when context was more strongly elaborated (i.e. in the high-context condition). The generally high proportion of ‘old’ responses to lure items indicates a strong reliance on familiarity-based judgments across conditions^55^, which may have limited the sensitivity of the task to detect more subtle effects of contextual elaboration on mnemonic discrimination.

Notably, participants in the low-context condition showed a higher rate of incorrect ‘new’ responses to lures. This pattern may be explained by reduced familiarity-based evidence in the absence of contextual elaboration. Without substantial contextual cues, lure items may have failed to evoke a familiarity signal strong enough to support an ‘old’ or ‘similar’ classification, resulting in a higher likelihood of responding ‘new’. This interpretation is further supported by the shorter encoding duration observed in the low-context condition, which may have limited the accumulation of familiarity-based strength^55,73^.

Importantly, the higher rate of responding ‘new’ to lures does not contradict the superior target recognition observed in the low-context condition. Target recognition and lure discrimination place different demands on memory signals: familiarity-based evidence may suffice to distinguish studied targets from novel items, yet remain insufficient to discriminate highly similar lure items. Consistent with models in which familiarity reflects a graded strength signal evaluated relative to competing representations^54^, lure items may fall below the decision threshold under low-context encoding despite intact target recognition. However, some limitations should be acknowledged when interpreting this pattern. First, familiarity was not directly measured, and second, as noted above, contextual reinstatement was not assessed, constraining interpretation of the underlying processes.

Contrary to our initial assumption, childhood adversity was not associated with a broad impairment in mnemonic discrimination. Neither adversity nor encoding condition alone significantly predicted performance, suggesting that discrimination abilities are relatively preserved across encoding demands in the present task. Given the overall low discrimination accuracy and the strong reliance on familiarity-based responses to lure items across conditions, potential effects of childhood adversity on lure discrimination may have been difficult to detect at the behavioral level. Accordingly, the present data do not support a robust association between childhood adversity and mnemonic discrimination under the current task demands.

Taken together, these findings raise important theoretical and potential clinical implications for understanding how childhood adversity related to subtle variations in memory performance. From a clinical perspective, such subtle, task-dependent differences in memory performance may reflect early cognitive alterations that contribute to vulnerability for adverse mental health outcomes^59,74^. Although the present study does not allow conclusions about underlying neural mechanisms, the results underscore the importance of considering task demands and contextual support when assessing memory function in relation to early life stress. Future research combining behavioral paradigms with neuroimaging approaches may help clarify how such subtle behavioral differences relate to broader neural systems involved in memory processing. Moreover, as our sample consisted of healthy adults with relatively low overall trauma exposure, the generalizability of the findings to other forms of early adversity or clinical populations remains limited. Nevertheless, the CTQ has been widely validated in both clinical and non-clinical samples, and the distribution of adversity on the subscale level in our study aligns with previous reports, supporting the robustness of our findings in this domain^36,75^.

In conclusion, we show that contextual elaboration during encoding does not uniformly benefit recognition memory performance, and may, under certain task conditions, be associated with reduced object recognition performance. Rather than reflecting deficits in contextual memory, the present results underscore the importance of task demands, attentional allocation, and the diagnostic value of contextual information for recognition outcomes. With respect to childhood adversity, the findings indicate a descriptive association with object recognition performance that was more apparent under conditions of reduced contextual support, without evidence for differential effects across encoding conditions. Overall, these results suggest that associations between early life stress and memory performance may be subtle and task-dependent, even in healthy adults. Future studies integrating behavioral and neurobiological measures will be necessary to further clarify the mechanisms underlying these associations and their relevance for long-term mental health outcomes.

## Electronic Supplementary Material

Below is the link to the electronic supplementary material.

Supplementary Material 1

## Data Availability

Data will be made available upon reasonable request to the corresponding author, including background images used in the experiment.
