# Supplementary material for: Contextual elaboration shapes object recognition memory across levels of childhood adversity in healthy adults
Source: Sci Rep. 2026 May 18;16:15343. doi: 10.1038/s41598-026-53083-5 (PMC13184324; doi:10.1038/s41598-026-53083-5)
Supplement: Supplementary file 1 — Supplementary Material 1 [file 41598_2026_53083_MOESM1_ESM.docx]

**Supplementary material**

**Table S1.** Between-group comparison of response proportions across item types and response categories

| Item type | Response type | High-context M (SD) | Low-context M (SD) | *t(df)* | *p* | 95%CI | *d* |
| --- | --- | --- | --- | --- | --- | --- | --- |
| Target | Old | 77.09 (17.40) | 85.79 (9.90) | 2.70 (60.87) | 0.009* | [2.25, 15.15] | 0.61 |
|  | Similar | 17.29 (13.45) | 10.99 (8.68) | -2.44 (65.40) | 0.017 | [-11.46, -1.14] | -0.55 |
|  | New | 5.62 (7.68) | 3.23 (3.72) | -1.75 (55.57) | 0.086 | [-5.15, 0.35] | -0.39 |
| Lure | Old | 52.26 (16.03) | 49.30 (13.48) | -0.87 (74) | 0.389 | [-9.74, 3.83] | -0.20 |
|  | Similar | 40.67 (16.64) | 39.70 (14.89) | -0.27 (74) | 0.790 | [-8.20, 6.26] | -0.06 |
|  | New | 7.07 (7.07) | 11.00 (8.40) | 2.21 (74) | 0.015* | [0.38, 7,47] | 0.51 |
| Foil | Old | 2.22 (3.27) | 2.25 (3.52) | 0.04 (74) | 0.969 | [-1.52, 1,58] | 0.01 |
|  | Similar | 10.77 (9.25) | 9.01 (7.37) | -0.91 (74) | 0.367 | [-5.63, 2.10] | -0.21 |
|  | New | 87.01 (9.82) | 88.73 (8.55) | 0.69 (74) | 0.416 | [-2.49, 5.95] | 0.19 |

Note. For all between-group comparisons involving response categories (‘old’, ‘similar’, ‘new’), Bonferroni correction was applied (k = 3), yielding an adjusted significance level of α = .0167.

**Table S2.** Relative prevalence of exposure across CTQ subscales in both conditions

| CTQ | High-context % (n) | Low-context % (n) | *Χ² (df=1)* | *p* |
| --- | --- | --- | --- | --- |
| Emotional abuse | 41.0 (16) | 37.8 (14) | 0.08 | .776 |
| Emotional neglect | 46.2 (18) | 45.9 (17) | <0.001 | .985 |
| Physical abuse | 0 (0) | 5.4 (2) | 2.17 | .234^a^ |
| Physical neglect | 23.1 (9) | 43.2 (16) | 3.50 | .061 |
| Sexual abuse | 15.4 (6) | 18.9 (7) | 0.17 | .683 |
| Sum Score | 46.2 (18) | 48.6 (18) | 0.05 | .828 |

Note. Participants were classified as exposed to childhood adversity if their subscale scores met or exceeded the ‘low to moderate’ threshold based on Bernstein et al. [43]: Emotional abuse ≥ 9, Emotional neglect ≥ 10, Physical abuse ≥ 6, Physical neglect ≥ 8, Sexual abuse ≥ 6. As no validated cut-off scores exist for the sum score, a median split for a classification of relatively elevated levels of exposure was used (≥ 35). ^a^Fisher’s Exact test

Table S3. Overview of regression models including main effects and condition × CTQ interaction

| **Memory Score** | **CTQ predictor** | **Term** | **R² base** | **R² full** | **ΔR²** | **β** | **SE** | **t** | **p** | **95%CI** |
| --- | --- | --- | --- | --- | --- | --- | --- | --- | --- | --- |
| ORM | CTQ sum score | Intercept | 0.112 | 0.126 | 0.014 | 0.33 | 0.11 | 3.11 | .003 | [0.12; 0.54] |
| ORM | CTQ sum score | condition |  |  |  | -0.61 | 0.23 | -2.68 | .009 | [-1.06; -0.16] |
| ORM | CTQ sum score | CTQ sum score (z-standardized) |  |  |  | -0.25 | 0.10 | -2.43 | .017 | [-0.45; -0.04] |
| ORM | CTQ sum score | Interaction |  |  |  | 0.26 | 0.26 | 1.00 | .319 | [-0.26; 0.78] |
| ORM | Emotional neglect | Intercept | 0.099 | 0.128 | 0.029 | 0.30 | 0.11 | 2.88 | .005 | [0.09; 0.52] |
| ORM | Emotional neglect | condition |  |  |  | -0.58 | 0.22 | -2.62 | .011 | [-1.03; -0.14] |
| ORM | Emotional neglect | Emotional neglect (z-standardized) |  |  |  | -0.25 | 0.09 | -2.64 | .010 | [-0.44; -0.06] |
| ORM | Emotional neglect | Interaction |  |  |  | 0.36 | 0.23 | 1.55 | .125 | [-0.10; 0.81] |
| ORM | Emotional abuse | Intercept | 0.101 | 0.112 | 0.011 | 0.32 | 0.11 | 2.94 | .004 | [0.10; 0.53] |
| ORM | Emotional abuse | condition |  |  |  | -0.60 | 0.22 | -2.67 | .009 | [-1.05; -0.15] |
| ORM | Emotional abuse | Emotional abuse (z-standardized) |  |  |  | -0.22 | 0.09 | -2.44 | .017 | [-0.39; -0.04] |
| ORM | Emotional abuse | Interaction |  |  |  | 0.21 | 0.23 | 0.95 | .346 | [-0.24; 0.66] |
| LDI | CTQ sum score | Intercept | 0.004 | 0.011 | 0.007 | 0.04 | 0.16 | 0.24 | .815 | [-0.28; 0.36] |
| LDI | CTQ sum score | condition |  |  |  | -0.05 | 0.24 | -0.22 | .830 | [-0.53; 0.43] |
| LDI | CTQ sum score | CTQ sum score (z-standardized) |  |  |  | -0.11 | 0.16 | -0.68 | .498 | [-0.43; 0.21] |
| LDI | CTQ sum score | Interaction |  |  |  | 0.18 | 0.28 | 0.65 | .519 | [-0.38; 0.75] |
| LDI | Emotional neglect | Intercept | 0.016 | 0.017 | <0.001 | 0.03 | 0.16 | 0.18 | .860 | [-0.29; 0.35] |
| LDI | Emotional neglect | condition |  |  |  | -0.05 | 0.24 | -0.22 | .823 | [-0.53; 0.43] |
| LDI | Emotional neglect | Emotional neglect (z-standardized) |  |  |  | -0.14 | 0.14 | -1.03 | .305 | [-0.41; 0.13] |
| LDI | Emotional neglect | Interaction |  |  |  | 0.04 | 0.28 | 0.15 | .880 | [-0.51; 0.59] |
| LDI | Emotional abuse | Intercept | 0.002 | 0.072 | 0.069 | 0.04 | 0.16 | 0.24 | .809 | [-0.28; 0.36] |
| LDI | Emotional abuse | condition |  |  |  | -0.03 | 0.23 | -0.13 | .895 | [-0.50; 0.43] |
| LDI | Emotional abuse | Emotional abuse (z-standardized) |  |  |  | -0.17 | 0.14 | -1.21 | .231 | [-0.45; 0.11] |
| LDI | Emotional abuse | Interaction |  |  |  | 0.54 | 0.22 | 2.46 | .016 | [0.10; 0.98] |

Note. ORM = Object recognition memory, LDI = Lure discrimination index. All regression coefficients are based on HC3 robust standard errors.

Table S4. Overview of mixed-effects logistic regression models predicting recall responses

| Item category | Response type | Predictor | β | SE | z | p | OR | 95% CI OR |
| --- | --- | --- | --- | --- | --- | --- | --- | --- |
| Target | Old | Intercept | 2.18 | 0.22 | 9.96 | 0 | 8.81 | [5.74; 13.52] |
|  |  | Encoding duration (z) | -0.05 | 0.1 | -0.49 | .624 | 0.95 | [0.78; 1.16] |
|  |  | Condition | -0.66 | 0.25 | -2.64 | .008 | 0.52 | [0.32; 0.84] |
|  |  | Encoding duration × Condition | 0.27 | 0.14 | 1.92 | .055 | 1.31 | [0.99; 1.71] |
|  | Similar | Intercept | -2.49 | 0.21 | -11.86 | <.001 | 0.08 | [0.05; 0.13] |
|  |  | Encoding duration (z) | 0.05 | 0.11 | 0.41 | .682 | 1.05 | [0.84; 1.30] |
|  |  | Condition | 0.63 | 0.24 | 2.61 | .009 | 1.87 | [1.17; 3.00] |
|  |  | Encoding duration × Condition | -0.24 | 0.15 | -1.59 | .112 | 0.79 | [0.59; 1.06] |
|  | New | Intercept | -3.82 | 0.29 | -13.28 | <.001 | 0.02 | [0.01; 0.04] |
|  |  | Encoding duration (z) | 0.07 | 0.17 | 0.41 | .683 | 1.07 | [0.76; 1.51] |
|  |  | Condition | 0.39 | 0.34 | 1.15 | .249 | 1.47 | [0.76; 2.84] |
|  |  | Encoding duration × Condition | -0.19 | 0.24 | -0.79 | .432 | 0.83 | [0.51; 1.33] |
| Lure | Old | Intercept | -0.11 | 0.26 | -0.42 | .674 | 0.9 | [0.54; 1.48] |
|  |  | Encoding duration (z) | -0.08 | 0.08 | -1 | .318 | 0.92 | [0.78; 1.09] |
|  |  | Condition | 0.20 | 0.18 | 1.1 | .271 | 1.22 | [0.86; 1.72] |
|  |  | Encoding duration × Condition | 0.00 | 0.12 | 0.03 | .979 | 1 | [0.80; 1.27] |
|  | Similar | Intercept | -0.48 | 0.22 | -2.12 | .034 | 0.62 | [0.40; 0.96] |
|  |  | Encoding duration (z) | 0.06 | 0.08 | 0.77 | .442 | 1.07 | [0.90; 1.26] |
|  |  | Condition | -0.02 | 0.19 | -0.12 | .904 | 0.98 | [0.67; 1.42] |
|  |  | Encoding duration × Condition | 0.16 | 0.12 | 1.37 | .171 | 1.18 | [0.93; 1.48] |
|  | New | Intercept | -2.49 | 0.23 | -11.03 | <.001 | 0.08 | [0.05; 0.13] |
|  |  | Encoding duration (z) | 0.06 | 0.12 | 0.48 | .634 | 1.06 | [0.84; 1.33] |
|  |  | Condition | -0.56 | 0.24 | -2.32 | .020 | 0.57 | [0.36; 0.92] |
|  |  | Encoding duration × Condition | -0.69 | 0.22 | -3.15 | .002 | 0.5 | [0.33; 0.77] |
| Foil | Old | Intercept | -4.54 | 0.41 | -11.19 | <.001 | 0.01 | [0.00; 0.02] |
|  |  | Encoding duration (z) | -0.28 | 0.29 | -0.95 | .344 | 0.76 | [0.43; 1.34] |
|  |  | Condition | -0.02 | 0.42 | -0.05 | .961 | 0.98 | [0.43; 2.23] |
|  |  | Encoding duration × Condition | 0.44 | 0.36 | 1.22 | .221 | 1.55 | [0.77; 3.11] |
|  | Similar | Intercept | -2.7 | 0.23 | -12.01 | <.001 | 0.07 | [0.04; 0.10] |
|  |  | Encoding duration (z) | 0.14 | 0.13 | 1.11 | .269 | 1.16 | [0.89; 1.49] |
|  |  | Condition | 0.16 | 0.23 | 0.68 | .496 | 1.17 | [0.74; 1.84] |
|  |  | Encoding duration × Condition | -0.09 | 0.17 | -0.51 | .608 | 0.92 | [0.66; 1.27] |
|  | New | Intercept | 2.39 | 0.21 | 11.67 | <.001 | 10.96 | [7.33; 16.38] |
|  |  | Encoding duration (z) | -0.07 | 0.12 | -0.59 | .554 | 0.93 | [0.73; 1.18] |
|  |  | Condition | -0.13 | 0.22 | -0.6 | .548 | 0.88 | [0.58; 1.34] |
|  |  | Encoding duration × Condition | -0.01 | 0.16 | -0.06 | .952 | 0.99 | [0.73; 1.34] |


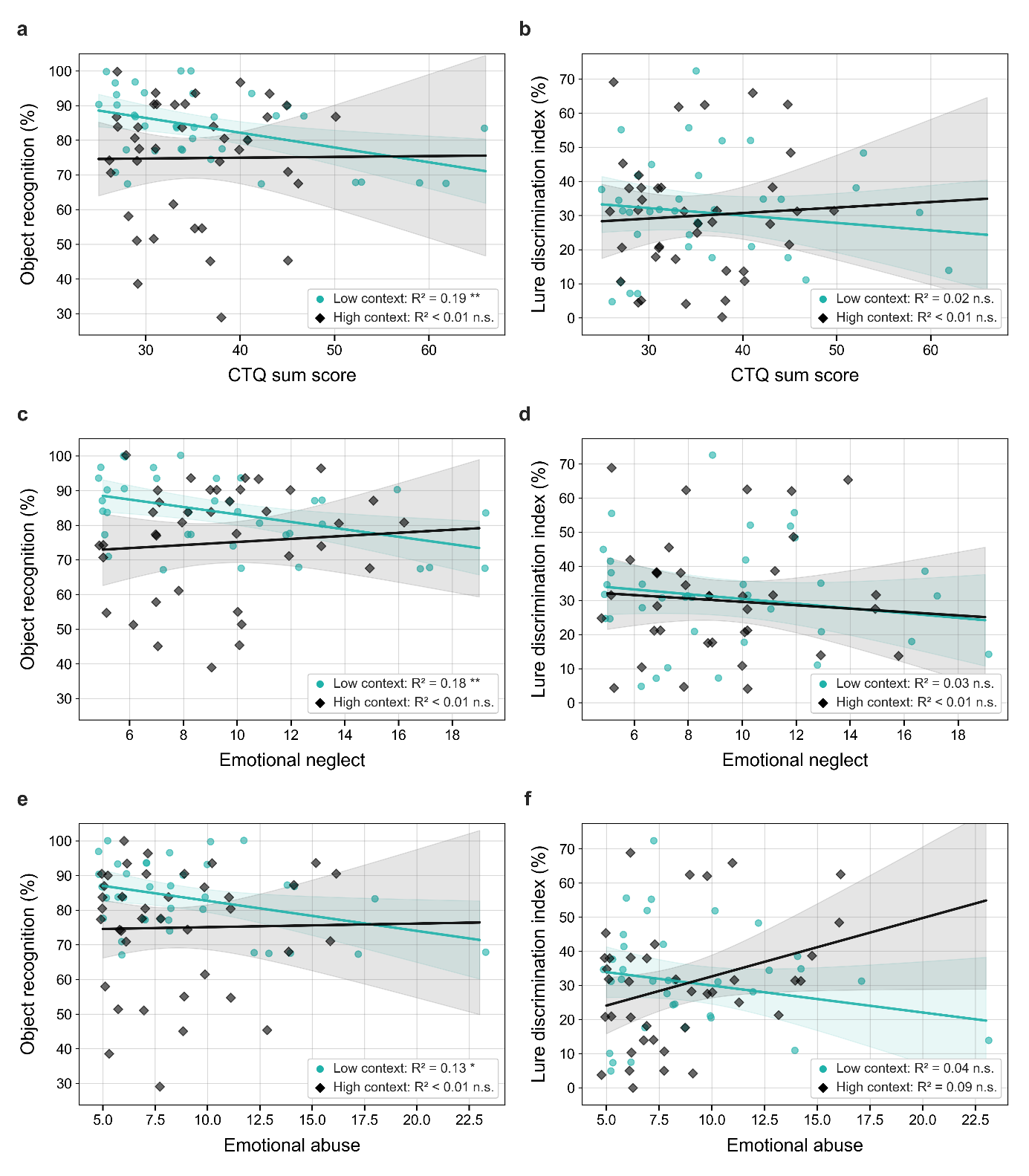


**Figure S1.** Scatter plots depicting the relationship between childhood adversity measures and memory performance indices. Significant correlations are marked accordingly (**p < 0.01, * p < 0.05). Slopes represent fitted regression lines for each encoding condition and are shown for descriptive purposes only, as the corresponding interaction effects were not statistically significant. Shaded areas indicate 95% confidence intervals. Figure S1a is identical to Figure 3.
